# A commentary on GST reforms and their effect on cancer care in India

**DOI:** 10.3389/fpubh.2026.1758151

**Published:** 2026-02-04

**Authors:** Vaibhav Sahni, Abhishek Shankar

**Affiliations:** Department of Radiation Oncology, All India Institute of Medical Sciences, New Delhi, India

**Keywords:** cancer, GST, India, low and middle income countries (LMIC), tax, tobacco

## Introduction

The cancer treatment pathway can tend to be ill-defined in a number of settings in low- and middle-income countries (LMICs) such as India. This scenario is compounded by issues pertaining to both affordability and access to care. In what should be considered significant economic steps toward battling cancer, the government of India has implemented a string for reforms to the Goods and Services Tax (GST) structure of the country several of which impact cancer care both directly and indirectly (1). In light of the sheer ambit of these policy changes along with the impact of their implementation on cancer care in India, the authors deemed it appropriate to provide an appraisal of the suggested changes as well as an opinion on their ramifications.

## Methods

The authors performed an analysis of literature available in the public domain regarding the recent GST reforms in India. Given the fact that these changes are quite recent, there was no original research data available to qualify the real-world ramifications of these changes. The authors restricted the search to news sources, both government and private in order to delineate the ambit of policy changes set to come in to effect at the time. Only data from mainstream news outlets or official government sources was included and appraised. News articles in both English and Hindi languages were utilized. The initial search was conducted in September 2025 and the last rendition of the search was done in December 2025 in order to ensure that the literature was current. All claims made in private news sources were cross-analyzed from official government releases and were also chronologically evaluated for any changes between September to December 2025.

## Discussion

The GST council in its 56th meeting has recommended the total exemption of 33 life-saving drugs including those used in cancer care from GST altogether (erstwhile 12% to now zero and three critical drugs for rare diseases and cancer from 5% earlier to zero) (1).

The council has also recommended no GST application on individual health and life insurance policies (from 18% previously) (1). An important aspect of this policy reform is that it includes certain patented drugs with no generic alternatives. A list of the pertinent drugs is provided in the Table 1.

**Table 1:** List of drugs with GST reduced to nil as part of the GST 2.0 reforms.

**S. No**.	**Drug name**	**Application of the drug**
1	Agalsidase beta	Enzyme replacement therapy
2	Imiglucerase	
3	Eptacog alfa activated recombinant coagulation factor VIIa	Hemophilia/factor VII deficiency treatment
4	Onasemnogene abeparvovec	Spinal muscular atrophy
5	Asciminib	Chronic myeloid leukemia
6	Daratumumab	Multiple myeloma
7	Pegylated liposomal irinotecan	Metastatic pancreatic adenocarcinoma
8	Mepolizumab	Severe asthma, chronic rhinosinusitis with nasal polyps, eosinophilic granulomatosis, hyper-eosinophilic syndrome
9	Alectinib	ALK positive non-small cell lung cancer
10	Amivantamab	Non-small cell lung cancer with EGFR mutation
11	Teclistamab	Multiple myeolma
12	Daratumumab subcutaneous	Multiple myeloma
13	Atezolizumab	Urothelial carcinoma, non-small cell lung cancer, hepatocellular carcinoma, melanoma
14	Entrectinib	Non-small cell lung cancer
15	Polatuzumab vedotin	B-cell lymphoma
16	Risdiplam	Spinal muscular atrophy
17	Idursulphatase	Hunter syndrome
18	Rurioctocog alpha pegol	Hemophilia
19	Agalsidase alpha	Fabry disease
20	Spesolimab	Pustular psoriasis
21	Emicizumab	Hemophilia
22	Avelumab	Merkel cell carcinoma
23	Tepotinib	Non-small cell lung cancer
24	Olipudase alpha	Acid sphingomyelinase deficiency
25	Laronidase	Mucopolysaccharidosis I
26	Inclisiran	Primary hyperlipidemia
27	C1-inhibitor injection	Angioedema
28	Cystamine bitartrate	Nephropathic cystinosis
29	Evolocumab	Cardiovascular disease, primary hyperlipidemia, hypercholesterolemia
30	Alirocumab	Cardiovascular disease, primary hyperlipidemia, hypercholesterolemia
31	Velmanase alfa	Alpha-mannosidosis
32	Miglustat	Gaucher disease
33	Belumosudil	Chronic graft vs. host disease

In another significant step, the council has also increased the tax slab for tobacco products to 40% which is the highest for any class of goods in the country (1). Tobacco and products related to it shall however, remain under the 28% tax slab till such time as loans and compensation cess have been paid (2). Regardless, the new taxation slab on health-harming products is a step in the right direction and provides increased opportunity to redirect the generated revenue for funding cancer care in the country.

There is evidence in literature which supports the fact that tobacco taxation leads to improved health outcomes particularly in economically disadvantaged sections of society (3).

The benefits of such taxation include gain in life years, averting treatment costs and premature mortality as well as avoiding catastrophic expenditure on health and poverty (3).

There is however an acknowledged paucity of evidence for the benefits of health taxation in terms of alcohol and sugar-sweetened beverages (3). Previous work has suggested policy reforms in the taxation regime (3).

A subnational study using the extended-cost effectiveness model on four Indian states found that a cigarette price hike of INR (Indian National Rupee) 10 with ad valorem of 10% (currently in four states of India led to 65,762 top-income and 485,725 bottom income bracket quitters for smoking thereby avoiding 665,000 mortalities, gaining 11.9 million years of life, over USD (United States Dollar) $1.96 trillion in averted treatment cost and saving over USD $762.5 million for AB-PMJAY (Ayushman Bharat Pradhan Mantri Jan Arogya Yojana) wherein the modeling factored only below poverty coverage of AB-PMJAY (India's universal health coverage scheme) (3).

Recent literature suggests that a 50% hike in tobacco tax stands to avert 1.8 million mortalities while saving, over the span of a decade, INR 11.9 trillion (4).

Literature has acknowledged that affordability for cigarettes has either remained neutral or improved and that for smokeless tobacco has improved in India over a period of time and increasing the tax slab for these is a welcome step (5).

High-cost drugs set to become GST free (provided a complete pass-through fraction is realized) include Pegylated Liposomal Irinotecan, Asciminib (USD $2568.36 for 60 capsules), Polatuzumab (USD $2425.94 per injection), Alectinib (USD $5372.81: 224 capsule pack), Amivantamab (USD $950.10), Teclistamab (USD $4220.16 per 1.7 mL injection), Daratumumab (USD $813.58 per 400 mg infusion) and Atezolizumab (current cost with 12% GST: INR 396,725/USD $4501.59 per 1,200 mg vial) amongst others.

The pricing is as per the Tata 1 mg pharmacy portal accessed in September 2025 (6). For a single 1,200 mg vial of Azetolizumab, assuming a complete pass-through fraction, the patient stands to save INR 42,506.25. For perspective, as per a People's Research on India's Consumer Economy (PRICE) report, destitutes earn less than INR 125,000 per annum per household, aspirers INR 125,000–500,000 and the middle-class INR 500,000–3,000,000 (7).

All costs are current with 12% GST applicable and conversion rate current for September 2025. In fact, all other medicines and drugs have seen a slashed GST to 5% from the earlier 12% (8, 9).

Economically, this tends to portend an “inverted GST” structure wherein manufacturers might end up with greater input tax compared to that on the final product. The key starting materials and active pharmaceutical ingredients have had their 18% taxation remain unchanged which creates increased chances tax inversion despite a provisional refund to the tune of 90% income tax credit. This scenario may be mitigated as long as the processing of claims is smooth (9).

The key starting materials and active pharmaceutical ingredients have had their 18% taxation remain unchanged which creates increased chances tax inversion despite a provisional refund to the tune of 90% income tax credit.

This will also ensure that manufacturers are able to pass on the benefits to consumers in a timely manner. From an industry perspective, the clear 5% slab across the board obviates ambiguity and enables clarity in marketing and transparency in pricing. This can aid in re-directing resources to research and innovation (10).

GST has also been reduced on diagnostic and surgical equipment as well as medical supplies such as bandages and gauze.

## Limitations

This appraisal of literature intends to provide an opinion on recent policy changes and their possible ramifications in healthcare for India. By this virtue, the data to form this opinion was derived from news sources albeit mainstream in case of private outlets and government outlets which officially notify the policy change. The obvious limitation is the dearth of original research data on the matter which will require time to be produced. Another limitation is that the realized pass-through fraction to the consumer will be reflected in time though government sources have urged industry to pass the complete benefits of the tax rationalization to the consumer.

## Conclusion

Overall, these economic policy changes should be regarded as steps in making cancer care more affordable, accessible as well as ones which promote health in society. The key takeaways from these reforms in terms of healthcare involve a simplified tax structure, removal of tax on drugs and medical equipment and increased taxation on tobacco products. There needs to be further research to have an accurate assessment of the predicted decrease in financial burden for cancer-afflicted households as well as the degree to which this will be blunted by universal health coverage under the Ayushman Bharat Pradhan Mantri Jan Arogya Yojana (AB-PMJAY). The aforementioned structural policy changes can also serve as a guidance for other countries in the region with similar socio-economic and disease burden characteristics which might benefit from adopting or adapting these measures in terms of policy layout, tax structure and outcome monitoring.
